# TIM-3 as a Target for Cancer Immunotherapy and Mechanisms of Action

**DOI:** 10.3390/ijms18030645

**Published:** 2017-03-16

**Authors:** Wenwen Du, Min Yang, Abbey Turner, Chunling Xu, Robert L. Ferris, Jianan Huang, Lawrence P. Kane, Binfeng Lu

**Affiliations:** 1 Department of Respiratory Medicine, The First Affiliated Hospital of Soochow University, Suzhou 215006, China; daisy910917@163.com (W.D.); huang_jian_an@163.com (J.H.); 2 Department of Immunology, School of Medicine, University of Pittsburgh, EBST E1047, 200 Lothrop Street, Pittsburgh, PA 15261, USA; carolyangmin@163.com (M.Y.); alt126@pitt.edu (A.T.); ferrrl@upmc.edu (R.L.F.); lkane@pitt.edu (L.P.K.); 3 Department of Immunology, School of Biology and Basic Medical Science, Soochow University, Suzhou 215123, China; 4 Department of Ophthalmology, The Second Hospital of Jilin University, Changchun 130041, China; xucl@jlu.edu.cn

**Keywords:** TIM-3, T cell subsets, tumor microenvironment, antitumor immune responses

## Abstract

Cancer immunotherapy has produced impressive clinical results in recent years. Despite the success of the checkpoint blockade strategies targeting cytotoxic T lymphocyte antigen 4 (CTLA-4) and programmed death receptor 1 (PD-1), a large portion of cancer patients have not yet benefited from this novel therapy. T cell immunoglobulin and mucin domain 3 (TIM-3) has been shown to mediate immune tolerance in mouse models of infectious diseases, alloimmunity, autoimmunity, and tumor Immunity. Thus, targeting TIM-3 emerges as a promising approach for further improvement of current immunotherapy. Despite a large amount of experimental data showing an immune suppressive function of TIM-3 in vivo, the exact mechanisms are not well understood. To enable effective targeting of TIM-3 for tumor immunotherapy, further in-depth mechanistic studies are warranted. These studies will also provide much-needed insight for the rational design of novel combination therapy with other checkpoint blockers. In this review, we summarize key evidence supporting an immune regulatory role of TIM-3 and discuss possible mechanisms of action.

## 1. Introduction

Immune checkpoint inhibitors targeting cytotoxic T lymphocyte antigen 4 (CTLA-4) and programmed death receptor 1 (PD-1) have been proven highly effective in fighting cancer. Ipilimumab—a fully humanized cytotoxic T-lymphocyte–associate-d antigen 4 (CTLA-4) antibody—was the first immune checkpoint inhibitor associated with an improvement in overall survival in a phase 3 study involving patients with metastatic melanoma [1,2]. Approximately 20% of patients treated with Ipilumumab had long-term survival [3,4]. Evidence suggests at least part of the mechanism of action of CTLA-4 monoclonal antibody (mAbs) functions by depleting regulartory T cells (Tregs) [5,6]. PD-1 is a key checkpoint molecule expressed in exhausted CD8^+^ T cells and Tregs. Blockade of PD-1 has achieved revolutionary clinical impact in many solid cancers [7,8,9,10,11,12,13,14,15,16,17,18]. Despite these encouraging results, large portions of cancer patients fail to respond to these therapies. One likely roadblock is the compensatory immune inhibitory pathways. Therefore, there is an urgent need to further improve these therapies by simultaneously targeting multiple immune checkpoint pathways.

T cell immunoglobulin and mucin domain 3 (TIM-3) has been recognized as a member of the TIM gene family, which includes TIM-1, TIM-3, TIM-4 in humans and Tim-1-8 in mice. TIM-3 is expressed on Th1, Th17, CD8^+^ T cells–cells of myeloid lineages [19,20,21] in mice. Engagement between TIM-3 and its ligands has been found to suppress Th1 and Th17 responses [22] and induce peripheral immune tolerance [23,24], supporting an inhibitory role of TIM-3 in T cell-mediated immune responses. TIM-3 expression also characterizes exhausted T cells during chronic infection [25,26,27,28]. In this setting, TIM-3-expressing CD4^+^ and CD8^+^ T cells produce reduced amounts of cytokine or are less proliferative in response to antigen [25,26,27,28]. Experiments with TIM-3 mAbs suggest that it plays a functional role in exhausted T cells. TIM-3 mAbs restores proliferation and enhances cytokine production in HIV-1-specific T cells in vitro [25]. Administration of TIM-3 and PD-1 mAbs synergistically control tumor growth [29,30,31,32]. These preclinical studies implicate an inhibitory role of TIM-3 in antitumor Immunity. However, the underlying mechanisms are not well understood and seemingly even contradictory to its well established T cell-stimulating function [33].

Herein, we summarize key evidence indicating an immune regulatory role of TIM-3. We further discuss possible mechanisms of action by which TIM-3 regulates immune responses in infectious immunity, autoimmunity, and tumor Immunity. We will discuss feasible approaches for targeting TIM-3 for cancer immunotherapy.

## 2. Molecular Characteristics of TIM-3, Its Ligands, and Signal Transduction

**Figure 1:**
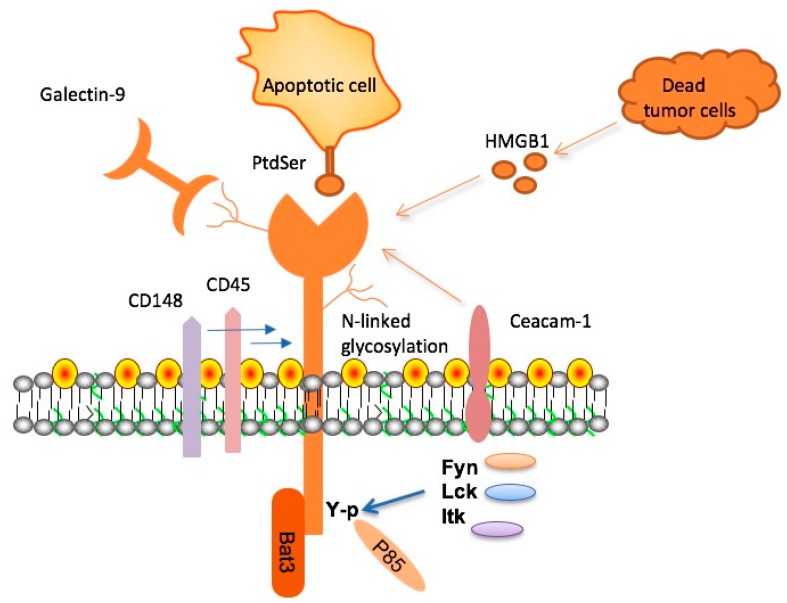
T cell immunoglobulin and mucin domain 3 (TIM-3), its ligands, and signaling adaptor proteins. Four ligands—namely, galectin-9 (Gal-9), phosphatidylserine (PtdSer), high mobility group protein B1 (HMGB1), and carcinoembryonic antigen cell adhesion molecule 1 (Ceacam-1)—have been identified to bind to the variable immunoglobulin (IgV) domain of TIM-3. In terms of signaling, HLA-B associated transcript 3 (Bat-3) binds to the cytoplasmic tail of TIM-3 and inhibits TIM-3 function. Fyn, Lck, and Itk, three tyrosine kinases, bind and phosphorylate specific tyrosine residues within the cytoplasmic domain of TIM-3. The phosphorylated tyrosines within the cytoplasmic domain of TIM-3 can recruit other downstream signaling adaptors such as p85 adaptor protein. In addition, Gal-9 can mediate the formation of clusters containing TIM-3, CD45, and CD148.

The TIM family is comprised of type I membrane proteins, which share a similar structure: a variable immunoglobulin domain (IgV), a glycosylated mucin domain of varying length in the extracellular region, and a single transmembrane domain [34,35,36,37,38,39]. With the exception of Tim-4, all TIM molecules also contain a C-terminal cytoplasmic tail with a conserved tyrosine-based signal motif [34,35,36,37,38,39]. There are six cysteines within the IgV domain of TIM-3, which makes this IgV domain different from others. Most TIM IgV regions contain four cysteines, which form two disulfide bridges that contribute to the formation of a unique binding surface [34]. In the classical IgV model, FG and CC’ loops are located at opposite ends of the domain, with a distance of 25 Å. However, in the TIM-3 protein, the CC’ loop is reoriented closer to the FG loop due to an additional disulfide bond [34,40]. Under this circumstance, a unique binding pocket (FG-CC′ cleft) is created, which is required for interactions with ligands of TIM-3 (Figure 1).

So far, four relevant ligands have been shown to interact with the IgV domain of TIM-3 (Figure 1) [41]. These include galectin-9 (Gal-9), high mobility group protein B1 (HMGB1), carcinoembryonic antigen cell adhesion molecule 1 (Ceacam-1), and phosphatidylserine (PtdSer). Gal-9, the first identified ligand, can bind to the carbohydrate motif on TIM-3 and incite an influx of calcium to the intracellular region of Th1 cells, inducing apoptosis [22]. It is important to note that glycosylation of the IgV is required for Gal-9 binding [22]. PtdSer, a molecule exposed on the surfaces of apoptotic cells, is another TIM-3 ligand. PtdSer was shown to bind to a pocket within the IgV domain of TIM-1, TIM-3, and TIM-4 [42]. This interaction facilitates the clearance of apoptotic bodies and also promotes the cross-presentation of antigens by dendritic cells (DCs) [43]. The third TIM-3 ligand identified is high mobility group protein B1 (HMGB1). TIM-3 is highly expressed on tumor infiltrating DCs and actively competes with nucleic acids released from dying tumor cells to bind HMGB1, effectively inhibiting stimulation of the innate immune response by nucleic acids [44]. As a result, activation of innate immunity and production of pro-inflammatory cytokines can be attenuated. Carcinoembryonic antigen cell adhesion molecule 1 (Ceacam-1) expressed on the cell surface is a more recently identified ligand for TIM-3. Ceacam-1 and TIM-3 are co-expressed, and the two molecules can form in *trans* a specific heterodimer which functions as a negative regulator of T cell responses [45]. The interaction between Ceacam-1 and TIM-3 can be either *cis* or *trans*, and both types of interactions can mediate T cell immune tolerance.

TIM-3 is involved in the proximal signaling events in T cells. However, there is evidence supporting both positive and negative effects of TIM-3 on TCR (T cell receoptor) signaling. Transient expression of TIM-3 increases TCR signaling to NFAT and NF-κB in Jurkat T cells and phosphorylation of tyrosines 256 and 263 is required, but the ecto domain of TIM-3 is not required for this function of TIM-3 [36]. Several members of the Src kinase family—such as Lck, Fyn and Itk—have been shown to bind and phosphorylate TIM-3 tyrosines [36,46]. The phosphorylated tyrosines within the cytoplasmic domain of TIM-3 can then recruit p85 adaptor protein, leading to the activation of PI3 kinases. TIM-3 has also been shown to inhibit TCR proximal signaling [47,48]. In TIM-3^+^ CD8^+^ effector T cells, Gal-9 is shown to induce co-localization of TIM-3 with receptor phosphatases CD45 and CD148, and thereby inhibits TCR signaling [49]. Another report shows that TIM-3 is stably expressed in Jurkat cells inhibits TCR-mediated NF-κB/NFAT activation [49]. Bat-3 has been shown to bind to the cytoplasmic tail of TIM-3, and Gal-9-triggered phosphorylation of tyrosines 256 and 263 leads to its release from TIM-3 [47]. Bat-3 reverses the negative effect of TIM-3 on TCR signaling, likely by recruiting active Lck or blocking the binding of Fyn to TIM-3 [41,47]. Therefore, the effect of TIM-3 on TCR signaling is dependent on cellular context (positive for naïve/effector/memory T cells versus negative for exhausted T cells) or the ligands it engages.

## 3. Tim-3 Mediates Immune Tolerance in Autoimmunity and Alloimmunity

In earlier studies, TIM-3 was shown to be involved in the regulation of autoimmune diseases in both mouse models and human patients. Treatment with TIM-3 mAb enhances the severity of experimental autoimmune encephalomyelitis (EAE) [21]. Lower expression of TIM-3 on T cells and higher production of interferon (IFN)-γ can be detected in patients suffering from multiple sclerosis (MS) [50]. TIM-3-Ig fusion protein or anti-TIM-3 mAb can accelerate autoimmune diabetes by blocking TIM-3-mediated immune tolerance [23]. These studies suggest that TIM-3 is an immune checkpoint molecule. Similar observations were also made in the transplantation setting. Administration of the TIM-3-Ig fusion protein prevented transplantation tolerance, and this function of TIM-3 is dependent on donor-specific CD4^+^CD25^+^ regulatory T cells [24]. Many other studies further support a critical role of TIM-3 in suppressing the rejection of transplanted skin [24], pancreatic islets [24], heart [51], and bone marrow allografts [52]. These studies indicate that TIM-3 signaling—or at least the immune cells expressing TIM-3—mediates immune tolerance. However, the in vivo mechanisms are still not clear.

## 4. TIM-3 in Immune Tolerance to Tumors

Recently, TIM-3 has been shown to be highly expressed on tumor antigen-specific T cells in the peripheral blood and among tumor infiltrating lymphocytes (TIL), suggesting a role of TIM-3 in tumor Immunity. Up-regulation of TIM-3 is associated with the exhaustion of tumor antigen-specific CD8^+^ T cells in melanoma patients, and administration of TIM-3 mAbs can reverse tumor-induced T cell exhaustion [29,53]. In human patients with non-small cell lung cancer (NSCLC), TIM-3 is predominantly expressed in tumor-infiltrating CD4^+^ and CD8^+^ T cells, but expressed at minimal levels on T cells from peripheral blood. In addition, among CD4^+^ TIL, TIM-3 is preferentially expressed on Foxp3^+^ CD4^+^ Treg cells, and the frequency of CD4^+^TIM-3^+^ TIL is correlated with poor patient survival [54]. TIM-3 was also shown to be expressed in TIL or tumor antigen-specific T cells in peripheral blood from many cancer types such as hepatocellular cancer, cervical cancer, colorectal cancer, ovarian cancer [55], NSCLC [56,57], head and neck cancer [58], renal cell carcinoma (RCC) [59], gastric cancer [60], esophageal cancer [61], prostate cancer [62], and non-Hodgkin lymphoma [63].

In preclinical models, administration of TIM-3 mAbs alone has produced variable antitumor effects. TIM-3 has been shown to be upregulated in TIL in mouse tumor models such as CT26 colon adenocarcinoma, 4T1 mammary adenocarcinoma, and B16F10 melanoma [64]. On its own, administration of TIM-3 mAbs did not inhibit tumor growth in the CT26 model. However, combination of TIM-3 and PD-1 mAbs had a much greater antitumor effect than administration of TIM-3 or PD-1 mAbs alone [64]. Another study showed that TIM-3 mAbs injection slowed tumor progression in many mouse tumor models, such as MC38 colon carcinoma, WT3 sarcoma, CT26 colon adenocarcinoma, and TRAMP-C1 prostate tumor [65]. Furthermore, combination of TIM-3 mAbs and CTLA4 mAbs, or TIM-3 mAbs and PD-1 mAbs has had much greater antitumor effects [65]. These studies have established that targeting TIM-3 with monoclonal antibodies is a viable new immunotherapy for cancer. In addition, rational combinations of TIM-3 mAbs with the PD-1 mAbs and/or CTLA4 mAbs have great potential to further improve the current immunotherapeutic approaches to cancer. Currently, a Phase I-Ib/II open-label multi-center study of the safety and efficacy of TIM-3 mAbs as single agent and in combination with PD-1 mAbs in adult patients with advanced malignancies is ongoing (ClinicalTrials.gov Identifier: NCT02608268).

## 5. Targeting TIM-3 for Immunotherapy of Cancer

**Figure 2:**
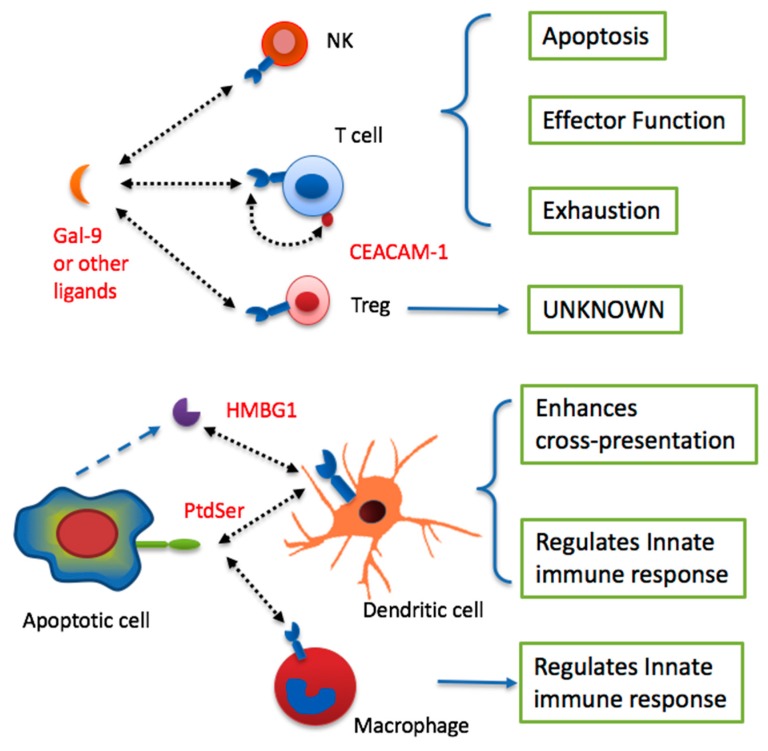
Summary of multiple biological functions of TIM-3 on various immune cells. TIM-3 signaling in T cells and NK cells leads to the development of effector functions, apoptosis, or exhaustion. The effect on Tregs remains unknown. Likely dependent on the cellular context, TIM-3 signaling can enhance cross-presentation of dendritic cells (DC) or inhibit innate immune responses of DC and macrophages.

TIM-3 signaling directly regulates the function of Th1 and CD8^+^ T cells through various mechanisms (Figure 2). TIM-3 is induced in Th1 cells and inhibits Th1-mediated immune responses by directly triggering apoptosis [22]. TIM-3 has also been identified as a marker for exhausted CD8^+^ T cells in patients with chronic infections such as HIV or hepatitis C virus [25,27]. In cancer patients, TIM-3 is upregulated on tumor antigen-specific CD8^+^ T cells and CD8^+^ TIL [29,53,54,64]. Administration of TIM-3 mAbs increases proliferation and cytokine production by tumor antigen-specific T cells [29,53,54,64,65]. These data support the idea that the exhaustion of T cells is involved in the establishment of an immune suppressive state in cancer.

However, one must be cautious when considering TIM-3 for the purpose of boosting CD8^+^ T cell-mediated immune responses. Despite the antitumor efficacy seen with TIM-3 mAbs, TIM-3 has been shown to promote CD8^+^ T cells function in the setting of listeria infection [33]. It is possible that although TIM-3 expression is associated with CD8^+^ T cell exhaustion during chronic infection and tumor immune suppression, it may also promote the initial generation of effector CD8^+^ T cells during acute infection.

TIM-3 is also found to be upregulated on CD4^+^ T cells in patients with chronic infection and cancer [25,27,54,64]. It is possible that TIM-3 is an exhaustion marker for Th1 cells [25,27]. It is important to note that a substantial proportion of CD4^+^TIM-3^+^ TIL are Foxp3^+^, suggesting a role for TIM-3 in Treg within the tumor microenvironment (TME) [64]. Besides tumor tissues, TIM-3 has also been found in many tissue-resident Tregs [66], and plays an important role in both maintaining immune tolerance as well as tissue repair [67,68,69,70]. It remains to be determined whether or not TIM-3 mAbs function in tumor immunotherapy—at least in part—by inhibiting the function of tumor-infiltrating Treg (Figure 2).

Ample evidence supports a role for TIM-3 as an inhibitory receptor on NK cells. TIM-3 is highly expressed on mature human NK cells and is variably expressed on immature NK cells [71]. TIM-3 marks NK cells with greater effector function, including cytokine production and cytotoxicity. However, cross-linking of TIM-3 inhibits NK cell-mediated cytotoxicity, suggesting that interaction of TIM-3 with one or more of its ligands negatively regulates NK cell activity [71]. In another study, TIM-3 expression was increased on NK cells from patients infected chronically with hepatitis B [72]. TIM-3 blockade ex vivo increased cytotoxicity of NK cells from these patients, suggesting that TIM-3 inhibits NK function in chronic infection. Many other studies further support an inhibitory role of TIM-3 in NK cell-mediated immune responses [73,74,75,76]. In contrast, some studies demonstrated a positive role of TIM-3 in NK function [77]. Therefore, TIM-3 might play both stimulatory and inhibitory roles in NK cells, depending on cellular and pathological context. The role of TIM-3 on NK cells in the tumor microenvironment remains to be examined (Figure 2).

Besides lymphocytes, TIM-3 has been shown to be expressed by various myeloid cells (e.g., DCs, monocytes, and macrophages), and to play an important role in regulating innate immune cell-mediated anti-viral infection and antitumor immune responses (Figure 2) [78]. TIM-3 is expressed on splenic CD8^+^ DCs [19,43]. Both Gal-9 and an agonistic TIM-3 mAb were reported to promote DC maturation and increase proinflammatory cytokine production [19,79]. TIM-3 mediates the uptake of apoptotic cells and the cross-presentation of antigen by DCs via TIM-3 binding to PtdSer [43]. TIM-3 mAbs inhibit this function of TIM-3 and inhibit cross-presentation of tumor antigens [43]. Despite evidence of a positive role for TIM-3 in DC function, other studies suggest that TIM-3 mediates inhibitory signals in DC, particularly in the TME. TIM-3 mAbs inhibited the activation and maturation of bone marrow-derived DCs by blocking the NF-κB pathway [80]. In tumor tissues, HMGB1—a ligand of TIM-3—is believed to act together with tumor-derived nucleic acids to gain access to endosomal vesicles and activate innate immune responses by DCs [81]. Through direct interaction with HMGB1, TIM-3—which is highly expressed on tumoral DCs—circumvents the stimulatory effects of nucleic acids in tumor immunity [81]. It is possible that the exact role of TIM-3 is dependent on the type of DC where it is expressed and the specific ligand through which it primarily engages these cells.

TIM-3 has been shown to be involved in both promoting and inhibiting the function of monocytes and macrophages. It was reported that antibodies to TIM-3 led to increased activation of macrophages and aggravated autoimmune disease [21]. In the same vein, TIM3-specific mAbs and TIM-3 overexpression in macrophages inhibited TLR4-stimulated inflammatory cytokine production and promoted sepsis in vivo [82]. TIM-3 is constitutively expressed by primary CD14^+^ monocytes, and blockade of TIM-3 signaling or silencing of TIM-3 expression led to a significant increase in interleukin (IL)-12 and IL-10 production and a decrease in PD-1 [83,84]. These findings suggest that TIM-3 acts as a negative regulator of macrophages and monocytes during innate immune responses. In contrast, several studies indicated that interaction of Gal-9 and TIM-3 resulted in the activation of macrophages and promoted antibacterial activity, though still-undefined mechanisms [85,86,87,88].

The phenotype of myeloid cells is highly influenced by tumor progression. Tumor-associated macrophages (TAMs) and myeloid-derived suppressor cells are highly increased in the TME. TIM-3 is expressed on TAMs in a variety of tumors, including hepatocellular carcinoma, lung cancer, clear cell renal cell carcinoma, osteosarcoma, Langerhans cell sarcoma, and neoplasms derived from histiocytic and dendritic cells [89,90,91,92,93]. Interestingly, transgenic overexpression of TIM-3 driven by the CD2 promoter on some T cells resulted in an increase in systemic levels of CD11b^+^Ly-6G^+^ granulocytic myeloid-derived suppressor cells (MDSC) and inhibition of immune responses [94]. These data are consistent with a role for TIM-3 in immune suppression. Mechanisms by which TIM-3 affects the generation and function of TAMs and MDSCs remain to be elucidated.

## 6. Conclusions and Perspective

At present, TIM-3 is generally acknowledged as a negative regulator of antitumor Immunity. Several properties of TIM-3 make it an ideal target for the next generation of immunotherapy. For example, its selective expression on intratumoral T cells may allow for more precise therapy via the targeting of tumor-infiltrating T cells, potentially reducing non-specific toxicity. In addition, signaling downstream of TIM-3 is rather distinct from that of CTLA-4 and PD-1, which have reasonably well-defined inhibitory mechanisms. By contrast, TIM-3 can both enhance and inhibit proximal signaling in T cells, depending on the cellular context. Therefore, the distinctive expression and intracellular signaling suggest a great potential for targeting TIM-3 alone and in combination with current PD-1 and CTLA-4-based immunotherapy of cancer.

Accumulating data support the concept that TIM-3 blockade can increase cell-mediated antitumor immune responses. In addition, TIM-3 mAbs may synergize with other immunotherapy modalities to increase antitumor efficacy. However, due to the expression of TIM-3 in both myeloid cells and lymphocytes, the exact cellular mechanisms underlying the in vivo antitumor activity of TIM-3 mAbs are still not understood. This issue is particularly critical because TIM-3 has been shown to both promote and inhibit cellular immune responses, depending on the models used. A thorough understanding of the roles of different cell types that express TIM-3 in various tumors and tumor immunotherapy models is crucial for further drug development targeting TIM-3 and design of combination therapy with PD-1 or CTLA-4 mAbs.
